# Generation of brain tumours in mice by Cre-mediated recombination of neural progenitors *in situ* with the tamoxifen metabolite endoxifen

**DOI:** 10.1242/dmm.022715

**Published:** 2016-02-01

**Authors:** Anna Benedykcinska, Andreia Ferreira, Joanne Lau, Jessica Broni, Angela Richard-Loendt, Nico V. Henriquez, Sebastian Brandner

**Affiliations:** 1 Department of Neurodegenerative Disease, UCL Institute of Neurology, Queen Square, London WC1N 3BG, UK; 2 Division of Neuropathology, The National Hospital for Neurology and Neurosurgery, Queen Square, London WC1N 3BG, UK

**Keywords:** Brain tumour, Cre recombination, Endoxifen, *loxP* system, Tamoxifen

## Abstract

Targeted cell- or region-specific gene recombination is widely used in the functional analysis of genes implicated in development and disease. In the brain, targeted gene recombination has become a mainstream approach to study neurodegeneration or tumorigenesis. The use of the Cre-*loxP* system to study tumorigenesis in the adult central nervous system (CNS) can be limited, when the promoter (such as *GFAP*) is also transiently expressed during development, which can result in the recombination of progenies of different lineages. Engineering of transgenic mice expressing Cre recombinase fused to a mutant of the human oestrogen receptor (ER) allows the circumvention of transient developmental *Cre* expression by inducing recombination in the adult organism. The recombination of *loxP* sequences occurs only in the presence of tamoxifen. Systemic administration of tamoxifen can, however, exhibit toxicity and might also recombine unwanted cell populations if the promoter driving *Cre* expression is active at the time of tamoxifen administration. Here, we report that a single site-specific injection of an active derivative of tamoxifen successfully activates Cre recombinase and selectively recombines tumour suppressor genes in neural progenitor cells of the subventricular zone in mice, and we demonstrate its application in a model for the generation of intrinsic brain tumours.

## Abstract (precis)

**Summary:** An active tamoxifen derivative can be used for the *in vivo* induction of recombination *in situ* in mice by *CreERT2*. We show how this method can be used to generate brain tumours through recombination of tumour suppressor genes in the subventricular zone.

## INTRODUCTION

The adult central nervous system (CNS) contains several stem cell compartments, which have been studied extensively. One of the major regions in which new neurons and glial cells are generated is a germinal niche adjacent to the walls of the lateral ventricles in the adult brain (Doetsch, 2003; Doetsch and Alvarez-Buylla, 1996; Doetsch et al., 1999, 1997). The introduction of oncogenic mutations into this cell population has been used to model brain tumours, for example by Cre-mediated recombination of tumour suppressor genes such as *Pten*, *p53*, *Rb* or *Nf1* (Chow et al., 2011; Jacques et al., 2010; Zheng et al., 2008; Zhu et al., 2005). Although the Cre*-lox* system can avoid embryonic lethality hampering many classical gene knockout models, there are still limitations, as, for example, seen in transgenic mice expressing Cre recombinase under the control of the glial fibrillary acidic protein (*GFAP*) promoter. GFAP expression is not limited to astrocytes or to mature stem cells but is also transiently expressed in neural precursors during CNS development, resulting in a recombination of all progenies of cells that expressed Cre during development (Kwon et al., 2001; Lewandoski, 2001; Marino et al., 2002, 2000; Zheng et al., 2008; Zhuo et al., 2001). This can be circumvented by several approaches, for example by direct injection of a viral vector expressing Cre recombinase (Ahmed et al., 2004), which can be refined by a tissue-specific promoter such as *GFAP* (Jacques et al., 2010), or by using transgenic mice expressing *Cre* under the control of a tamoxifen-inducible promoter. Such mice express Cre recombinase fused to a mutated form of the human oestrogen receptor (ER), which results in the expression of the fusion protein CreERT2. In the inactive form, CreERT2 is quenched by heat shock 90 protein (Hsp90) and resides within the cytoplasm. Upon binding of the oestrogen analogue tamoxifen, CreERT2 is released from Hsp90 and is translocated into the nucleus, where it can catalyse the recombination of floxed DNA sequences. The generation of *CreERT2* transgenic mice allows the activation of *Cre* expression in a time-controlled fashion (Chow et al., 2008; Metzger and Chambon, 2001; Mori et al., 2006). Tamoxifen can induce Cre activity only after it is converted into its active metabolites, such as 4-hydroxytamoxifen (4-OH-TAM) or 4-hydroxy-N-desmethyltamoxifen (endoxifen) (Hayashi and McMahon, 2002). Topical application of tamoxifen to recombine *CreERT2*-driven promoters has been previously used for studies in skin biology. The limitations of some conditional knockout models of skin diseases are similar to those in the CNS, where complete and tissue-specific Cre-mediated gene knockout results in embryonic or early perinatal death, precluding the analysis of gene function in different cell types and in the regulation of skin homeostasis (Gunschmann et al., 2014; Lewandoski, 2001). In analogy to the more established trans-epidermal topical application of tamoxifen for *Cre*-mediated gene recombination, we set out to establish (i) whether injection of active metabolites into the cerebral ventricles can directly activate *Cre* expression in stem cells, thus targeting a regionally and spatially defined, specific population of cells and (ii) whether the regional expression in a selected cell population leads to the generation of tumours and how they might compare to tumours induced by recombination due to *Cre*-expressing adenovirus (Adeno-*Cre*) or Adeno*-GFAP-Cre* (Henriquez et al., 2013; Jacques et al., 2010). To this end, we used mice expressing *CreERT2* under control of the endogenous glutamate-aspartate transporter (*GLAST*) promoter (Mori et al., 2006), which is expressed in a range of glial cells including B-type stem cells (Doetsch, 2003; Doetsch and Alvarez-Buylla, 1996; Doetsch et al., 1999, 1997). By crossing these mice with conditional knockout mice carrying floxed alleles of the tumour suppressor genes *p53* (*p53^loxP/loxP^*) and *Pten* (*Pten^loxP/loxP^*), we explored whether this highly selective and regionally confined targeting would modify the phenotype of tumours arising from the subventricular zone (SVZ) stem/progenitor cells.

## RESULTS

### Optimisation of tamoxifen dosage for intracerebral injection

To establish an optimal concentration of 4-OH-TAM or endoxifen to elicit Cre expression with minimal toxicity, we sought to establish the following parameters: (i) optimal concentration for gene recombination, (ii) minimal toxicity and (iii) the optimal volume and carrier (DMSO or ethanol). Table 1 shows the volumes, concentrations and resulting amounts of drug injected intraventricularly. Because only *in vitro* data were available for the concentrations of 4-OH-TAM or endoxifen, we first set out to determine the volumes and concentrations of intraventricularly injected endoxifen and 4-OH-TAM required for *in vivo* cell recombination and to determine potential toxicity. This experiment would also inform about the occurrence of possible toxic side effects at higher concentrations.

**Table 1.**
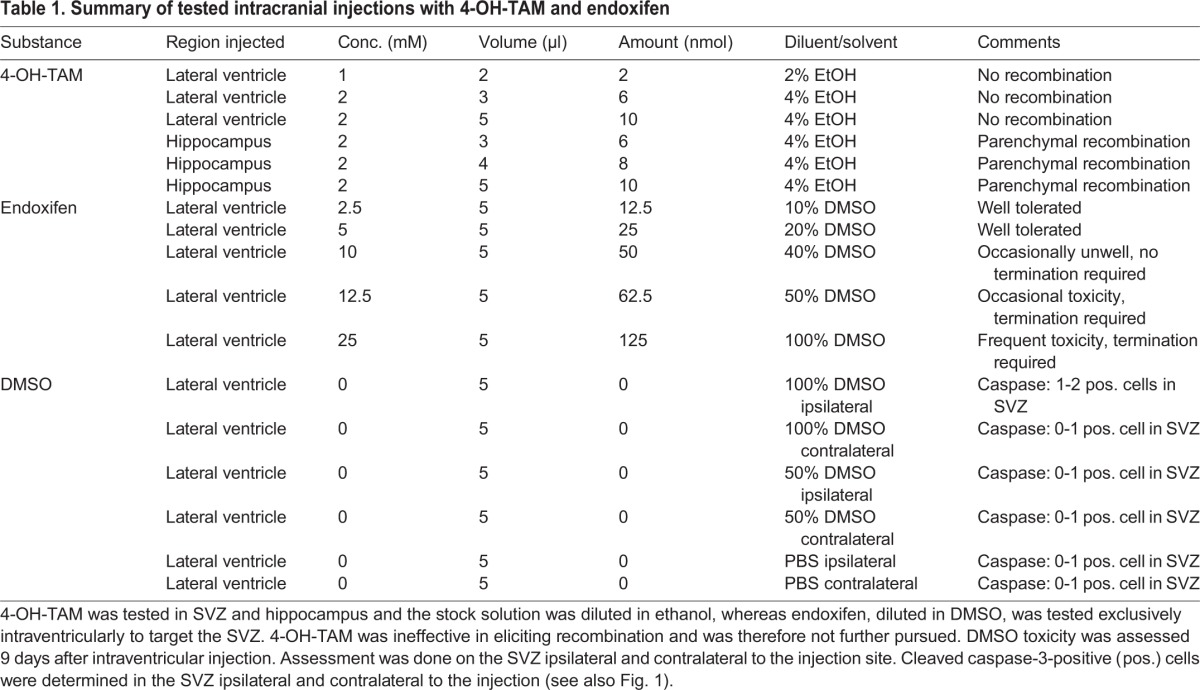
**Summary of tested intracranial injections with 4-OH-TAM and endoxifen**

**Fig. 1.**
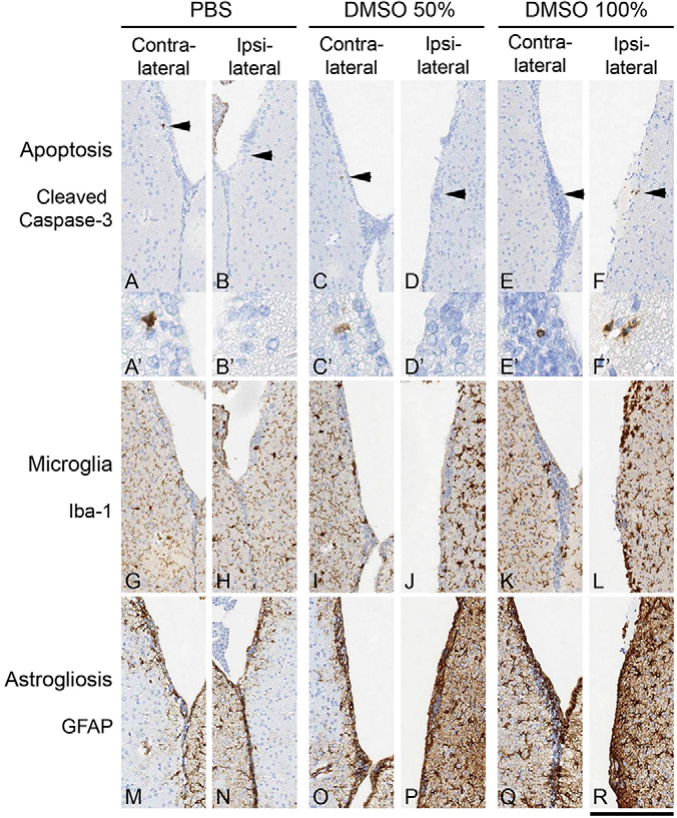
**Cellular reaction to intraventricular DMSO injection.** The cerebral ventricles were injected with 5 µl PBS (A,B,G,H,M,N), 50% DMSO in PBS (C,D,I,J,O,P) or 100% DMSO (E,F,K,L,Q,R). (A-F) Immunohistochemical staining for cleaved caspase 3 to detect apoptotic cells/nuclei shows occasional positive cells in controls and both DMSO groups. Arrowheads point to single positive cells in the SVZ, shown at higher magnification in A'-F'. Single cells are detected bilaterally in the SVZ. The PBS group shows a single cell contralateral to the injection (A), and there is a single cell in the contralateral wall in the 50% DMSO-injected brain, and a single cell on the contralateral side and two cells on the ipsilateral side of the 100% DMSO-injected brain. (G-L) Mice injected with DMSO show a mild microglial reaction on the injected side (J,L). (M-R) This is also associated with a mild to moderate astrocytic gliosis, which is present on the side of injection (P,R). Scale bar: 25 µm for A′-F′, and 100 µm in all other panels.

Although a 0.1 µM concentration of 4-OH-TAM is effective in cell culture experiments (Wassarman and Soriano, 2010), we started the dosage of intraventricular (ICV) injections at an arbitrary concentration of 1 mM dissolved in 2% ethanol/PBS (5 µl) to inject into the ventricles of *ROSA26^loxP/loxP^* mice, which we considered to be a compromise between minimal side effects and sufficient concentration at the ventricular wall in the circulating cerebrospinal fluid. However, recombination analysis of brains 1 week after injection showed no β-galactosidase activity and thus no recombination. Next, we injected 5 µl of 4-OH-TAM at a concentration of 2 mM dissolved in 4% ethanol into the hippocampus, which also contains *GLAST*-expressing cells. We regarded 4% ethanol as the maximum tolerable concentration in the brain. After a 1-week incubation, the brains were assayed for β-galactosidase activity and now showed recombination in the cortex, the hippocampus and the third ventricle, demonstrating that an active tamoxifen metabolite is capable of eliciting recombination in the context of a *CreERT2* transgenic system. However, the relatively ineffective recombination prompted us to further explore endoxifen (4-hydroxy-N-desmethyl-tamoxifen) (Ahmad et al., 2010; Johnson et al., 2004; Jordan, 2007; Stearns et al., 2003), an alternative derivative for which tamoxifen is metabolised in the liver and has so far not been used before for Cre-mediated induction of the *ERT2* system *in vivo*. We used concentrations of 2.5, 5, 10, 12.5 and 25 mM, dissolved in 10, 20, 40, 50 and 100% DMSO, respectively, corresponding to a drug dose of 12.5, 25, 50, 62.5 and 125 nmol (Table 1). No neurological signs of toxicity were observed at the 2.5 and 5 mM concentrations, whereas single-occurrence neurological signs suggestive of drug toxicity doses were observed at the 10 and 12.5 mM concentrations and more frequent neurological signs of toxicity at the 25 mM concentration were noted. No neurotoxicity was observed in mice that received up to 100% DMSO (vehicle) only, as reported previously (Blevins et al., 2002; Schick et al., 1990). To confirm the absence of morphologically detectable cellular damage, we assessed DMSO toxicity systematically by injecting 5 µl of PBS, 50% DMSO or 100% DMSO into the ventricle of *GLAST::CreERT2; ROSA26^lox^^P/loxP^* mice. All mice tested – the PBS-injected mice and the two groups of DMSO-injected mice – showed single cells positive for cleaved caspase3 (Fig. 1A-F) in the SVZ. There was no statistically significant increase of caspase in the DMSO-injected groups, which showed, as did controls, 0 or 1 caspase-3 positive cells in the SVZ. However, DMSO injection resulted in a mild increase of activated microglia (Fig. 1G-L) and astrocytes (Fig. 1M-R) on the side of the injection (microglia in Fig. 1J,P, and astrocytes in Fig. 1L,R).

### Endoxifen-mediated *CreERT2* recombination occurs in a dose-dependent fashion

**Fig. 2.**
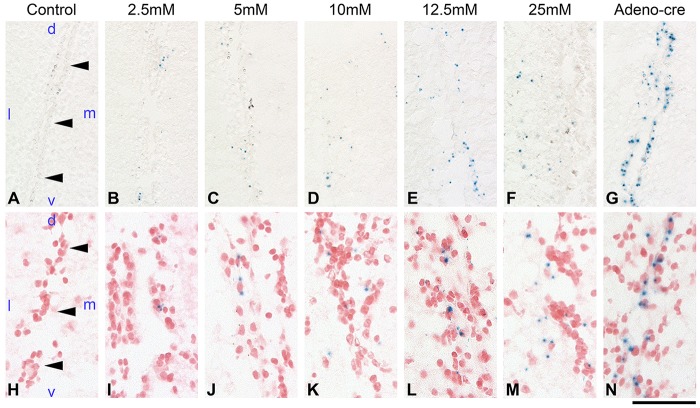
**β-galactosidase activity following endoxifen-mediated Cre-induced recombination in the SVZ of *GLAST::CreERT2;ROSA26^lox^^P/loxP^* reporter mice.** Coronal sections, developed for X-Gal enzymatic colourimetric reaction (A-G) and subsequent counterstaining with Nuclear Fast Red (H-N). (A,H) PBS-injected controls show no recombination. Arrowheads point to the lateral wall of the SVZ. d, dorsal; l, lateral; m, medial; v, ventral. (B-F,I-M) The number of X-Gal-positive cells in the SVZ increased with the concentration of endoxifen. (G,N) Adeno-*Cre*-mediated recombination shows a higher number of X-Gal-positive cells in the SVZ. Scale bar: 500 µm (A-G), 100 µm (H-N).

Recombination occurred at all concentrations of endoxifen tested, in a dose-dependent manner, as visualised by X-Gal staining in the SVZ (Fig. 2). At high endoxifen concentrations (12.5 mM and 25 mM), recombination took place in SVZ cells and rarely in adjacent, likely differentiated, astrocytes. At lower concentrations, recombined cells were restricted to the SVZ (Fig. 2A-D), and were also less frequent, thus reducing the number of potentially recombined, tumour-generating, cells in future experimental settings. Brains of non-injected or DMSO-injected *GLAST::CreERT2;ROSA26^lox^^P/loxP^* showed no recombination and thus no expression of β-galactosidase. Endoxifen-mediated Cre activation results in recombination of both alleles, as assessed by recombination PCR (see later) or by detection of pAkt, which becomes phosphorylated and accumulates in tumour cells in which PTEN protein expression is abolished (Groszer et al., 2001; Marino et al., 2002; also discussed later).

### Characterisation of cell types recombined by endoxifen in the brain of *GLAST::CreERT2;ROSA26^loxP/loxP^* mice

**Fig. 3.**
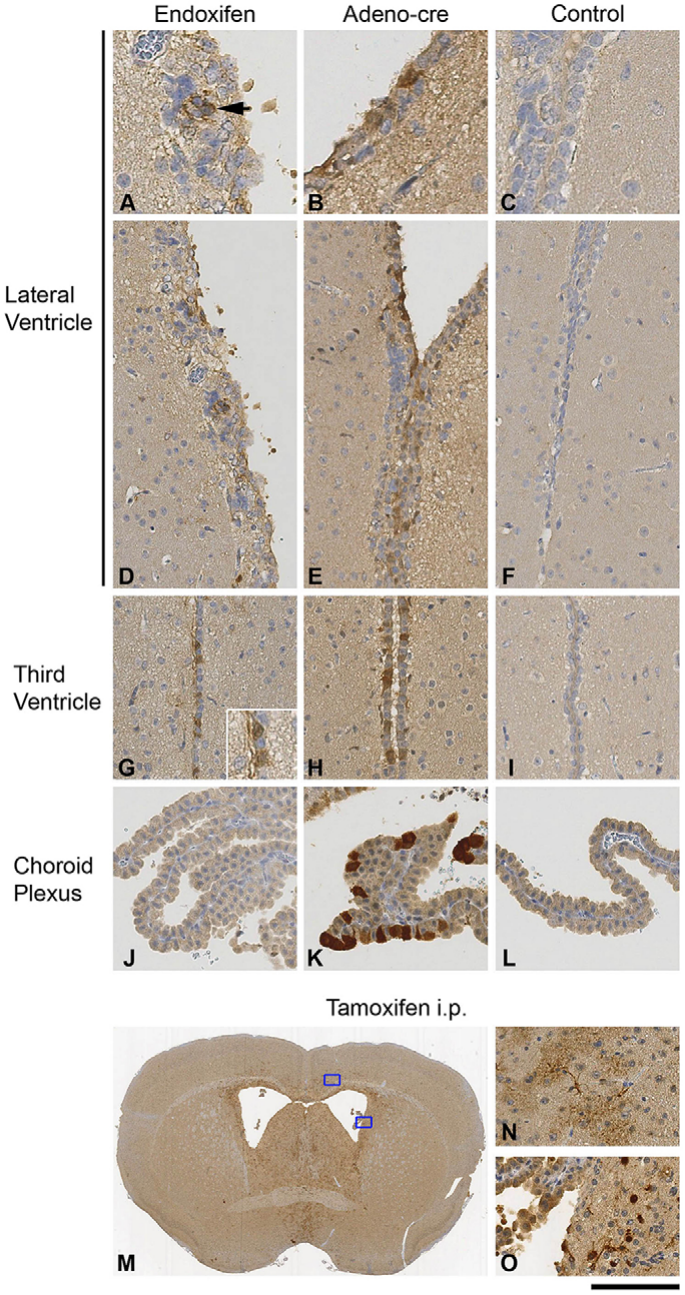
**Immunohistochemical detection of β-galactosidase expression in the brain of endoxifen-injected *GLAST::CreERT2;ROSA26^lox^^P/loxP^* or in Adeno-*Cre*-injected *ROSA26^lox^^P/loxP^* reporter mice.** Left column: expression of β-galactosidase in clusters of cells in the SVZ of the lateral ventricles (A,D), and in ependymal cells (G) of the third ventricle. Arrowhead in A points to a recombined cluster of cells. Cells of the choroid plexus (J) are not recombined: they do not express GLAST in this mouse. Insert in G shows recombined cells in the ependymal layer. Middle column: upon Adeno-*Cre*-mediated recombination, there is widespread expression of β-galactosidase in the cells of the SVZ (B,E), and ependymal cells of the third ventricle (H). Also, a population of choroid plexus cells (K) underwent recombination and expressed β-galactosidase. Right column: no recombination is seen in controls, which were injected with PBS. (M-O) Widespread expression in the brain is seen after intraperitoneal application of tamoxifen, including in astrocytes in the cortex (N; higher-magnification view of the uppermost box in M) and deep beneath the SVZ (O; higher-magnification view of the lowermost box in M). Scale bar: 50 µm (A-C,N,O), 100 µm (D-L), 1.8 mm (M).

Within the SVZ, GLAST is not expressed by B-type stem cells (Mori et al., 2006). We compared the endoxifen-induced Cre-mediated recombination with that of our established, and well-characterised, method of Adeno-*Cre*-mediated recombination, which served as control (Jacques et al., 2010). Immunohistochemical detection of β-galactosidase, a marker of recombination in *ROSA26^lox^^P/loxP^* reporter mice, shows widespread expression in the ventricular system, including the lateral (Fig. 3A,B,D,E) and the third (Fig. 3G,H) ventricles in both recombination methods, but not in the CNS parenchyma subjacent to the SVZ. Choroid plexus epithelial cells were recombined by Adeno-*Cre* (Fig. 3K), but not by the endoxifen (Fig. 3J) system because GLAST is not expressed by choroid plexus epithelium, confirming the specificity of the approach. As expected, intraperitoneal injection of tamoxifen base resulted in a widespread recombination in glial cells in the brain, including the SVZ (Fig. 3M-O), as previously reported (Mori et al., 2006), and controls were non-recombined throughout (Fig. 3C,F,I,L).

**Fig. 4.**
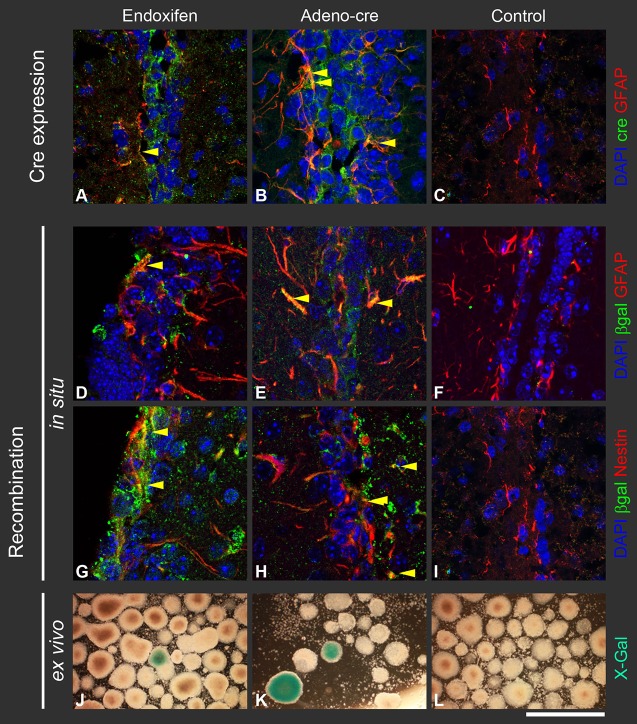
**Expression of Cre recombinase, and characterisation of recombined cells.** (A-C) Double-labelling immunofluorescence detects cells expressing Cre recombinase following endoxifen administration in *GLAST::CreERT2;ROSA26^lox^^P/loxP^* reporter mice (A) or following Adeno-*Cre*-mediated recombination in *ROSA26^lox^^P/loxP^* reporter mice (B). Co-expression of GFAP and Cre recombinase appears yellow. Arrowheads indicate cells expressing both antigens. (D-I) Recombination of (D-F) GFAP-expressing and (G-I) nestin-expressing cells in the SVZ indicates effective endoxifen-mediated recombination in *GLAST::CreERT2;ROSA26^lox^^P/loxP^* reporter mice and confirms targeting of GFAP- and nestin-expressing progenitors. Arrowheads indicate cells expressing both antigens. No Cre expression and recombination was observed in controls (C,F,I). (J-L) Stem/progenitor cells of the SVZ give rise to neurospheres when cultured in permissive medium. Stem/progenitor cells that underwent recombination *in vivo* by intraventricular injection of endoxifen (J) or Adeno-*Cre* (K) give rise to recombined, β-galactosidase-expressing, progenies, which form blue spheres in the X-Gal assay. No recombination was observed in controls (L). Scale bar: 50 µm (A-I), 300 µm (J-L).

Double-labelling immunofluorescence was used to colocalise the stem/progenitor marker GFAP with Cre-expressing cells (Fig. 4A-C) and the resulting recombination was assayed by detection of β-galactosidase (Fig. 4D,E,G,H). We found that a population of GFAP- and nestin-expressing cells in the SVZ showed colocalisation with β-galactosidase, indicating that the stem/progenitor cells, corresponding to B-type SVZ cells (Doetsch et al., 1999), underwent recombination with both methods.

### Recombination of *GLAST::Cre*-expressing SVZ stem/progenitor cells

Next, we sought to confirm that the cells that underwent recombination corresponded functionally and biologically to stem/progenitor cells, and were capable of forming neurospheres. Stem and progenitor cells from the SVZ can be cultured *in vitro* as neurospheres (Doetsch et al., 1999; Jacques et al., 1998, 2010; Lee et al., 2005; Soares and Sotelo, 2004). *GLAST::Cre(ERT);ROSA26^lox^^P/loxP^* mice were injected intraventricularly with 5 µl 5 mM endoxifen, and mice injected with 4 µl Adeno-*Cre* virus [10^9^ plaque-forming units (pfu)] served as positive control. At 7-10 days after ICV injection, mice were sacrificed and the SVZ dissected to isolate and derive stem and progenitor cells as described previously (Jacques et al., 1998). These stem/progenitor cells were then cultured in permissive medium (Jacques et al., 1998) to form neurospheres and assayed for recombination. *In vivo* recombination of neurogenic SVZ cells is thought to give rise to a clonally expanding population of recombined stem cells into a neurosphere, which can be quantified *in vitro* (Fig. 4J-L). We found in our unpassaged *in vitro* preparations derived from endoxifen-injected *GLAST::CreERT2;ROSA26^lox^^P/loxP^* mice that 1.9% of neurospheres showed recombination, compared to a higher rate of 4.3% of neurospheres in Adeno-*Cre*-recombined *ROSA26^lox^^P/loxP^* mice (Table 2).

**Table 2.**
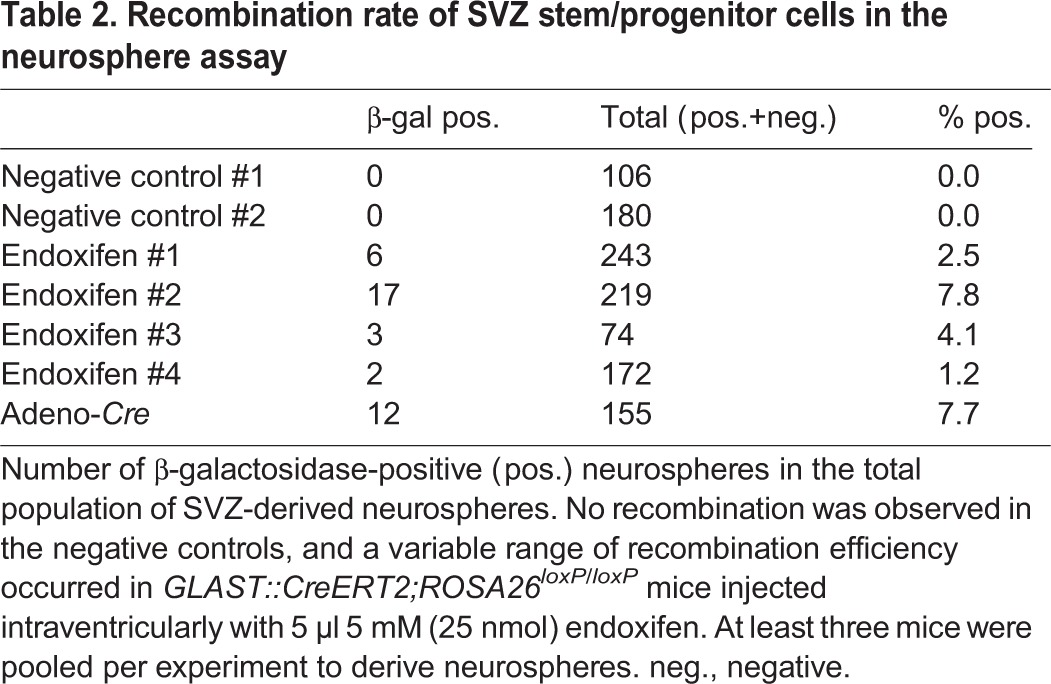
**Recombination rate of SVZ stem/progenitor cells in the neurosphere assay**

### Endoxifen-mediated recombination of the tumour suppressor genes *PTEN* and *p53* gives rise to brain tumours

**Fig. 5.**
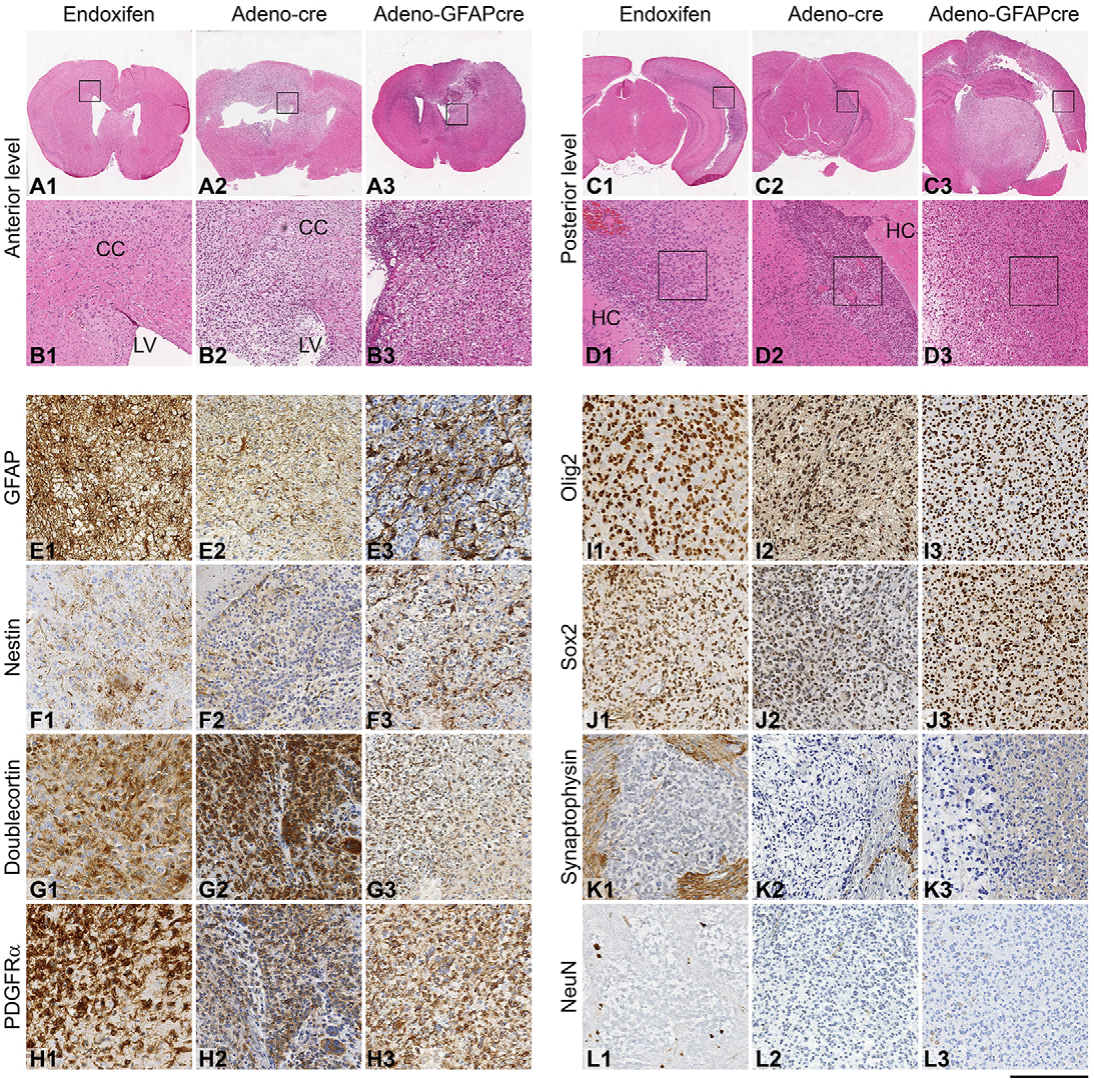
**Formation of gliomas.** The formation of gliomas was assessed in endoxifen-injected *GLAST::CreERT2;p53^loxP/loxP^;Pten^loxP/loxP^* mice (columns 1, 4), and in Adeno-*Cre*-injected (columns 2, 5) or Adeno-*GFAP*-*Cre*-injected *p53^loxP/loxP^;Pten^loxP/loxP^* mice (columns 3, 6). (A,C) Overview of coronal sections on the level of the SVZ. The boxes indicate the areas shown at higher magnification in B and D, showing a diffusely infiltrating glioma in the lateral corner of the ventricle. CC, corpus callosum; LV, lateral ventricle; HC, hippocampus. (C,D) Posterior level of the same brain as in A and B, showing an infiltrative tumour arising from the ventricle. The boxed areas in D indicate the areas shown in I-L. (E-L) Immunohistochemical analysis of the same brains, from the anterior level (A,B) or posterior level (C,D). Tumours elicited by the three methods show similar expression patterns, all consistent with gliomas. (E) Tumours express GFAP (note that underlying brain astrocytes also contribute to the GFAP-positive population). (F) Expression of nestin by tumour cell processes. (G-J) Strong expression of doublecortin (G), PDGFRα (H) and the markers Olig2 (I) and Sox2 (J). (K,L) None of the tumours express neuronal markers: synaptophysin (K) and NeuN (L) are negative in tumour cells, whereas they are expressed by surrounding brain tissue or in scattered entrapped neurones (L1). Scale bar: 4 mm (A,C), 500 µm (B,D) and 200 µm (E-L).

**Fig. 6.**
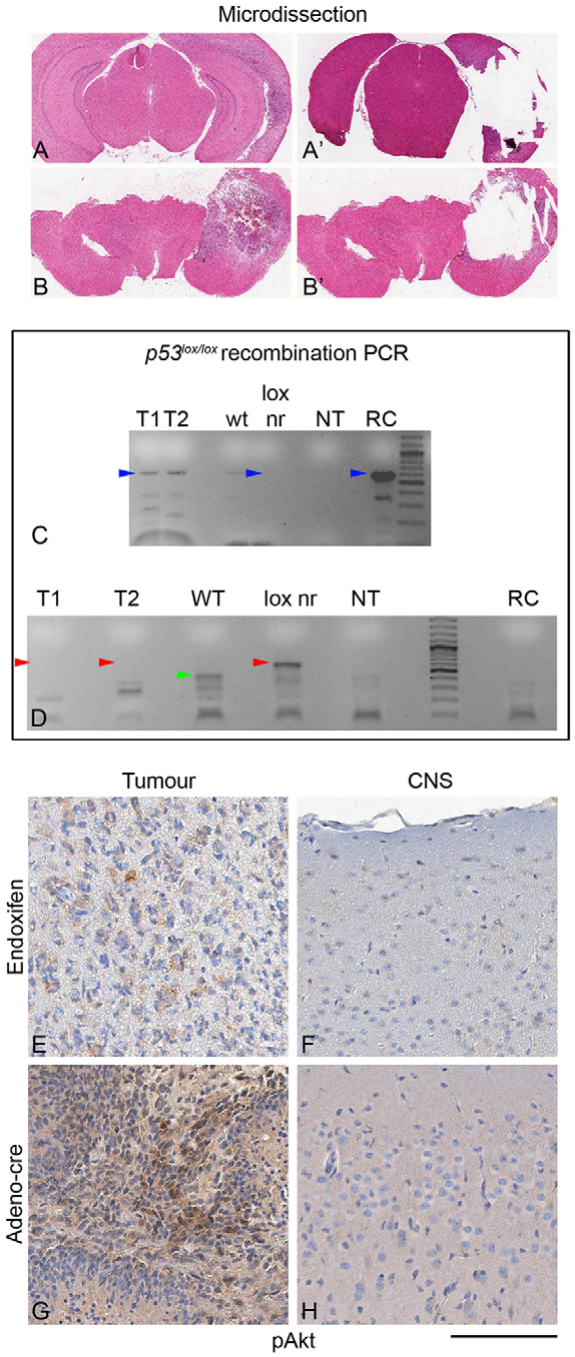
**Detection of gene recombination in endoxifen- or Adeno-*Cre*-induced tumours.** (A,B) Microdissection of tumours. Tumours correspond to T1 (A) and T2 (B) (see C). (A,B) Prior to microdissection; (A′,B′) after microdissection. (C) Recombination PCR showing a 612-bp band (blue arrowhead) in recombined tumour tissue (T1, T2) but not in wild-type (wt) brain, or non-recombined brain tissue of naïve *GLAST::Cre(ERT2);Pten^loxP/loxP^;p53^loxP/loxP^* mice (lox nr); NT, non-template control; RC, *in vitro*-recombined cell line derived from the SVZ of a *Pten^loxP/loxP^;p53^loxP/loxP^* mouse. (D) PCR detecting wild-type and non-recombined DNA only. A 512-bp band (red arrowheads) is seen only in non-recombined (lox nr) brain but not in recombined tumour (T1, T2). A 400-bp wild-type is seen in non-transgenic controls only. No signal is detected in the non-template control (NT) or in recombined *in vitro*-recombined cell line derived from the SVZ of a *Pten^loxP/loxP^;p53^loxP/loxP^* mouse. (E-H) Homozygous *Pten^loxP/loxP^* recombination (exon 5) leads to loss of functional PTEN protein and results in upregulation of pAkt (brown immunolabelling). (E,G) Tumour cells that undergo recombination of both alleles express detectable amounts of pAkt, whereas adjacent CNS tissue does not express pAkt (F,H). Scale bar: 100 µm in E-H.

We have previously shown that Adeno-*Cre-* or Adeno-*GFAP-Cre*-induced recombination of the tumour suppressor genes *Pten* and *p53* in stem/progenitor cells of the SVZ gives rise to glial tumours, with histological features of oligodendroglial and astrocytic tumours (Henriquez et al., 2013). Here, we tested whether the recombination of B-type stem/progenitor cells, using *GLAST::CreERT2* mice to recombine the tumour suppressor genes *Pten* and *p53* (*GLAST::CreERT2;Pten^loxP/loxP^;p53^loxP/loxP^*) would give rise to similar tumours. IVC injection of 25 nmol endoxifen in 5 µl volume generated tumours in three out of 16 injected mice. Histological analysis showed lesions of different sizes and with varying extents of infiltration. There were small neoplastic lesions arising from beneath the SVZ, extending into the striatum and dorsally into the corpus callosum. Larger tumours showed an expansion into and a diffuse infiltration of the entire caudate nucleus, and occasionally also infiltrated transcallosally into the contralateral hemisphere (Fig. 5). Histologically, the tumours showed a glial morphology with a range of features typical of astrocytomas and oligodendrogliomas (Fig. 5B,D). Up to 20 mitoses/10 high-power fields were observed, and some of the tumours contained areas of necrosis and occasional microvascular proliferations, similar to those in malignant gliomas in humans and similar to tumours generated by Adeno-*Cre* injection (Jacques et al., 2010). Endoxifen-induced tumours expressed GFAP, nestin, Sox2, Olig2, PDGFRa and doublecortin (Fig. 5E1-J1), and were indistinguishable from tumours generated by Adeno-*Cre* or Adeno-*GFAP-Cre* injection, as described previously (Henriquez et al., 2013; Jacques et al., 2010) and as shown in Fig. 5E2-J3. There was no expression of neuronal markers such as synaptophysin or NeuN in all three models (Fig. 5K,L). Homozygous recombination of both tumour suppressor genes was confirmed by microdissecting tumours (Fig. 6A,B) and by recombination PCR of floxed *p53* alleles (Fig. 6C,D), whereas homozygous recombination of the floxed *PTEN* alleles was assessed by the expression of pAkt (Fig. 6E,G), which becomes phosphorylated upon loss of function of both copies of *PTEN*, as described previously (Groszer et al., 2001; Kwon et al., 2001; Marino et al., 2002).

## DISCUSSION

We present here a method that allows for a cell-type-specific recombination, in a defined region of the brain, and validate its use to study the pathobiology of disease in a brain tumour model. Several different methods have been developed to achieve Cre-mediated recombination. Targeted gene inactivation often causes embryonic lethality, for example in knockout mice for *Rb* (Jacks et al., 1992), *Apc* (Su et al., 1992) or *Pten* (Di Cristofano et al., 1998; Stambolic et al., 1998; Suzuki et al., 1998). Subsequently, Cre-mediated inducible gene inactivation, combined with mice carrying *loxP* recognition sequences (Gu et al., 1994, 1993; Kuhn et al., 1995), was developed to circumvent embryonic lethality by targeting specific cell types or regions. Several possibilities exist to achieve the expression of Cre recombinase in a chosen cell type. Most commonly, a separate mouse line is generated that expresses Cre recombinase under the control of a cell- or tissue-specific promoter. An important caveat, essential for the correct interpretation of a phenotype, is that recombination begins once the *Cre* transgene becomes activated. During brain development, for example, many promoters, such as nestin, *GFAP* or engrailed-2 are transiently expressed in neural precursor/progenitor cell populations, resulting in a permanent deletion of the target gene, regardless of the fate of their progeny (Backman et al., 2001; Kwon et al., 2001; Marino et al., 2002, 2000). Topical application of Cre recombinase, for example using an adenoviral vector (Ahmed et al., 2004; Anton and Graham, 1995; Kaspar et al., 2002; Wang et al., 2006), is a successful approach to achieve recombination of *GFAP-* or nestin-expressing cells of the SVZ. Administered into the ventricles, the virus infects stem and progenitor cells of the SVZ, and also a few other cell types, such as choroid plexus epithelial cells. In the parenchyma, it recombines mature astrocytes and neurons (Jacques et al., 2010). A more selective expression in astrocytes was achieved by an adenovirus that expressed *Cre* under the control of the *GFAP* promoter (Jacques et al., 2010; Mirzadeh et al., 2008) (Fig. 5), but this reduced efficacy in eliciting brain tumours. An alternative approach is the use of tamoxifen-inducible Cre expression in the brain, for example *GFAP::CreERT* or nestin*::CreERT2* (Chow et al., 2011). Recombination in the *CreERT2* system is usually accomplished by intraperitoneal injection of tamoxifen, which is metabolised in the liver into metabolites that have a higher affinity to the mutated ER. Fused with Cre recombinase (Guo et al., 2002), this receptor is used in most ERT2 models, including the *GLAST::CreERT2* mouse. Systemic tamoxifen application, however, causes widespread additional recombination of astrocytes and Müller cells (Mori et al., 2006) (Fig. 3M-O). Instead, endoxifen, with its higher affinity to the mutated ER, injected into the ventricle of *GLAST::CreERT2* mice (Hayashi and McMahon, 2002; Lim et al., 2006; Stearns et al., 2003), restricted recombination to SVZ cells, likely to represent stem/progenitor cells (Figs 4A,D,G and 3A,D,G).

The limitations of this method are the toxicity of tamoxifen derivatives and thus the relatively smaller number cells that undergo recombination when using smaller dosages. Injection of 5 µl 25 mM endoxifen (125 nmol), dissolved in DMSO, caused a proportion of mice to exhibit neurological signs including seizures after a latency of 2-6 hours, requiring them to be culled. This toxicity is unlikely to be caused by DMSO because its injection as vehicle only did not cause side effects and showed no increased cell death (Fig. 1A,B). Reduction to 5 mM endoxifen concentration (dose 25 nmol) was tolerated well. However, recombination efficiency was correspondingly reduced (Fig. 2). This suggests that Cre-mediated recombination is a stochastic process, which has implications for tumorigenesis.

The objective to develop this model was to refine targeting of stem/progenitor cells using a cell-specific promoter (Mori et al., 2006) and an established combination of tumour suppressor genes, known to generate gliomas (Chow et al., 2011; Henriquez et al., 2013; Jacques et al., 2010; Zheng et al., 2008; Zhu et al., 2005). Here we compared an established, robust glioma model (Adeno-*Cre*-mediated recombination of *Pten^loxP/loxP^/p53^loxP/loxP^* in the SVZ), with that of endoxifen induced *GLAST::Cre-*mediated recombination of *Pten^loxP/loxP^/p53^loxP/loxP^*. The tumour incidence after endoxifen-induced recombination (3 of 13; 23%), was however similar to that in Adeno-*Cre*-induced mice (16 of 47; 34%) (*P*=0.52; Fisher's exact test) (Table 3). Tumours generated with all three approaches were histologically indistinguishable (Fig. 5), with a spectrum of astrocytic and oligodendroglial tumour features, similar to the variability in human tumours (Brandner and von Deimling, 2015), consistent with our previous findings (Henriquez et al., 2013; Jacques et al., 2010).

**Table 3.**
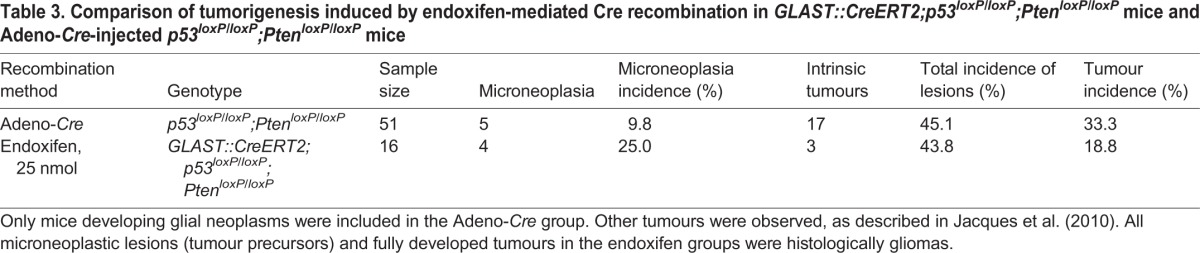
**Comparison of tumorigenesis induced by endoxifen-mediated Cre recombination in *GLAST::CreERT2;p53^loxP/loxP^;Pten^lo^^xP/loxP^* mice and Adeno-*Cre*-injected *p53*^*loxP/loxP*^*;Pten*^*loxP/loxP*^ mice**

**Fig. 7.**
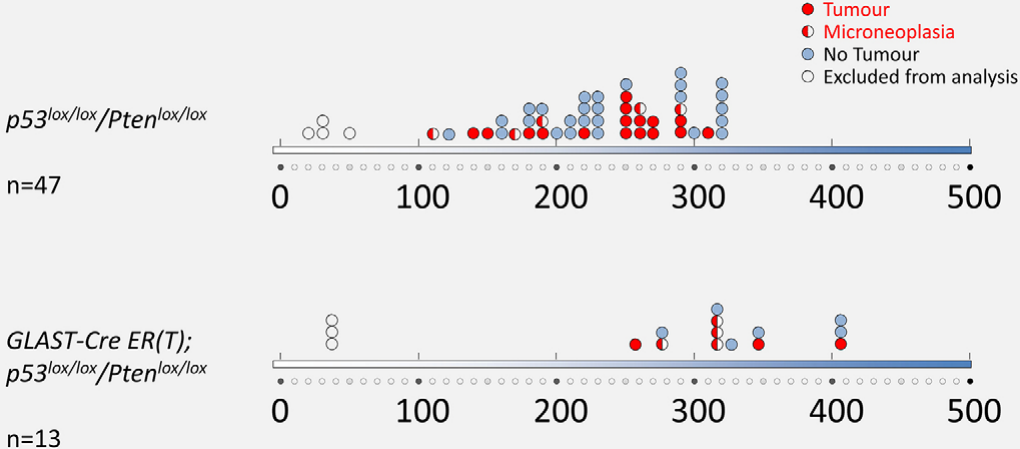
**Latency and frequency of tumour formation after Cre-mediated recombination.** The *x*-axis represents the latency in days and each circle represents an individual tumour. Half-filled circles represent precursor lesions. Mice that died prematurely or were culled owing to unrelated health concerns at 20-50 days of incubation were excluded from analysis, because tumour development does not occur before 100 days.

The target of Adeno-*Cre* is not precisely defined, and likely includes B-type stem cells and (at least a proportion of) C-type transient amplifying cells (Jacques et al., 2010), whereas recombination in the *GLAST::CreERT2* model is better defined, affecting chiefly B-type SVZ cells (Mori et al., 2006). The overall incidence of brain tumours does not show significant differences between the two models possibly because a recombination event in a restricted population (for example, B-type cells) will still give rise to progenies of these cells, and ultimately leads to a wider range of recombined progenies sufficient to form a brain tumour at a frequency that is comparable with that achieved with Adeno-*Cre*-mediated recombination (Fig. 7). However, there is a difference in latencies between these two models. To exclude that this was caused by incomplete recombination in the endoxifen model, we confirmed recombination of both alleles. Recombination-specific PCR (Marino et al., 2000) confirms that both *p53^loxP/loxP^* alleles underwent recombination (Fig. 6C,D) and, by detection of pAkt expression, that both *PTEN* alleles were recombined (Fig. 6E-H). Recombination of a single *PTEN* allele does not result in detectable pAkt expression (Marino et al., 2002), owing to the haplosufficiency of *PTEN*. Importantly, all three models, Adeno-*Cre*, Adeno-*GFAP-Cre* and endoxifen-induced *GLAST::CreERT2*-mediated recombination, elicit the same type of tumours, i.e. a range of astrocytic and oligodendroglial tumours with indistinguishable immunoprofiles (Fig. 6E-L). In conclusion, we have established and validated a novel approach to recombine a regionally defined cell population using direct application of the tamoxifen metabolite endoxifen in the CNS and we demonstrate its usefulness for specific biological questions, in that endoxifen-induced *GLAST::CreERT2* mediated recombination of tumour suppressor genes results in the formation of brain tumours.

## MATERIALS AND METHODS

### Transgenic mice

Combinations of the conditional mouse mutants *p53^loxP/loxP^* and *Pten^loxP/loxP^* (Marino et al., 2002, 2000) all in a *ROSA26^loxP/loxP^* background (Soriano, 1999) were used as described before (Henriquez et al., 2013; Jacques et al., 2010). *GLAST::CreERT2* mice were obtained from M. Goetz, Munich (Mori et al., 2006). Animals were kept according to institutional and UK Home Office guidelines (project licences 70/5540 and 70/6603). The ARRIVE guidelines were followed as part of the institutional policy and the licensing of the experiments.

### Intraperitoneal injection of tamoxifen

Animals were injected with 0.1 ml of 20 mg/ml tamoxifen each day for 5 days. Tamoxifen injectable solution was prepared with tamoxifen Free Base (Sigma T5648), ethanol and corn oil (Sigma C8267), as previously published (Lau et al., 2011; Madisen et al., 2010).

### Stereotaxic injection of adenovirus and tamoxifen metabolites

The *Cre* adenovirus vector was constructed and propagated as described (Akagi et al., 1997). Viral infection of SVZ progenitors was achieved by unilateral stereotaxic injections of 10^9^ pfu of adenovirus expressing Cre recombinase (in short Adeno-*Cre*) with a 26 G needle attached to a 10 µl gastight Hamilton syringe (model 1701 RN#80030) in 5 µl PBS into the left ventricle of compound mutant mice as described and characterised in detail previously (Jacques et al., 2010).

Tamoxifen was injected as active metabolite 4-hydroxytamoxifen (4-OH-TAM, SIGMA H7904) (Jordan, 2007), or 4-hydroxy-N-desmethyltamoxifen (endoxifen, Sigma E8284) (Ahmad et al., 2010; Johnson et al., 2004; Jordan, 2007; Stearns et al., 2003). 4-OH-TAM was dissolved at 1 mM concentration in 2% ethanol/PBS and 5 µl injected into the ventricles. Endoxifen had not been used before for the induction of the ERT2 system *in vivo*. Endoxifen hydrochloride hydrate (Sigma E8284) has a recommended solubility of over 10 mg/ml (∼25 mM) in 100% DMSO, corresponding to a maximum concentration of 25 mM or a dose of 125 nmol in a 5 µl volume. At this concentration, a proportion of mice developed neurological signs suggestive of neurotoxicity and had to be culled (see Results and Table 1). Subsequently, concentrations of 2.5, 5, 10, 12.5 and 25 mM, dissolved in 10, 20, 40, 50 and 100% DMSO, respectively, corresponding to a drug dose of 12.5, 25, 50, 62.5 and 125 nmol were used. For testing DMSO toxicity, groups of three mice were injected with 5 µl PBS, 50% DMSO in PBS or 100% DMSO in PBS. Mice were culled 9 days after injection and coronally sectioned brains were stained for cleaved caspase 3, GFAP and Iba1.

### Isolation, propagation and staining of neurospheres

Neurospheres were isolated from young adult mouse brains and propagated in serum-free medium based on DMEM/HamF12 (#D8437, Sigma), and supplemented with B27 (1:50; #17504-044, Invitrogen), EGF (20 ng/ml; #315-09, PeproTech), bFGF (20 ng/ml; #100-18B, PeproTech), as described previously (Jacques et al., 2010). For X-Gal enzymatic staining, neurospheres were transferred from the 10 cm dish into a four-well plate (well diameter 10 mm) with 150 µl growth medium, and incubated for 30 min in staining buffer that was composed of: 10 mM phosphate buffer, 150 mM NaCl, 1 mM MgCl_2_, 3.3 mM K_4_Fe(CN)_6_×3H_2_O, 3.3 mM K_3_Fe(CN)_6_, 1% MgCl_2_, 0.02% IGEPAL and 0.01% sodium deoxycholate and 1% X-Gal. Spheres were photographed through a Stemi SV11 (Zeiss) stereomicroscope, and counted using ImageJ.

### Histological examination and immunostaining

Mice that developed clinical signs of intracranial pressure were culled, and brain removed and fixed in 10% formalin, embedded in paraffin, cut to 3-µm sections and stained with haematoxylin-eosin (H&E). Immunostaining was done on Ventana Discovery automated staining machines (ROCHE, Burgess Hill, UK) following the manufacturer's guidelines, using horseradish-peroxidase-conjugated streptavidin complex and diaminobenzidine as a chromogen. The following antibodies were used for histological characterisation: anti-GFAP (DAKO Z0334, 1:1000), -NeuN (Chemicon MAB377, 1:1000), -nestin (Abcam ab11306, 1:400), -synaptophysin (Zymed 080130, prediluted), -Sox2 (Millipore AB5603, 1:100), -doublecortin (Abcam AB18723, 1:100), -Olig2 (Millipore AB9610, 1:100), -PDGFRα (Abcam ab15501, prediluted preparation), rabbit anti-β-galactosidase (Chemicon AB1211, 1:250), rabbit anti-Cre-recombinase (Covance PRB1061C, 1:500), anti-cleaved caspase-3 (Asp175) (Cell Signaling #9661); anti-phospho-Akt (Ser473) (D9E), and rabbit mAb (Cell Signaling #4060). Counterstain of fluorescent sections was done with 4′,6-diamidino-2-phenylindole, dihydrochloride (DAPI) (Life Technologies D1306).

For immunofluorescent antigen detection, cryostat sections of 14-µm thickness were prepared from perfusion-fixed, coronally oriented brains, and mounted on Superfrost Slides. Fluorescence-labelled secondary antibodies from molecular probes (Alexa Fluor 488, Alexa Fluor 546) were used for detection.

### Tissue microdissection, DNA extraction from paraffin sections and recombination PCR

PCR analysis of Cre-mediated recombination was performed on genomic DNA extracted from tumours microdissected from paraffin sections. DNA extracted from the cerebellum of an *En2cre; p53^loxP/loxP^* and *Pten^loxP/loxP^* mouse (similar to those described in Marino et al., 2002) and a cell line derived from *in vitro* recombined SVZ stem/progenitor cells of a *p53^loxP/loxP^* and *Pten^loxP/loxP^* mouse as described in Jacques et al. (2010) served as positive control.

MasterPure Complete DNA & RNA Purification Kit (Epicentre, Illumina) was used for extraction of DNA. Paraffin sections were de-waxed with xylene, followed by ethanol and air-dried. Microdissected tissue was digested with 4 μl proteinase K (50 μg/μl) in 300 μl of Lysis Solution and subsequent steps were carried out according to the manufacturer's instructions.

Reactions contained 30 ng of template DNA, 0.5 μm primers, 250 μm dNTPs, 9% glycerol, 2.5 units of FastStart Taq DNA polymerase (Roche) and 1× FastStart Taq PCR reaction buffer with 2 mM MgCl_2_ in a 50 μl volume. Thermocycling conditions consisted of 35 cycles of 30 s at 95°C, 60 s at 58°C, and 60 s at 72°C. *p53* recombination was assayed with primers 5′-CACAAAAACAGGTTAAACCCA-3′ (p53-int1-fwd) and 5′-GAAGA-CAGAAAAGGGGAGGG-3′ (p53-int10-rev) yielding a 612-bp product. The presence of the unrecombined *p53* floxed gene was assayed with primers 5′-AAGGGGTATGAGGGACAAGG-3′ (p53-int 10-fwd) and 5′-GAAGACAGAAAAGGGGAGGG-3′ (p53-int 10-rev) yielding a 584-bp product (unrecombined floxed *p53* allele) and/or 400-bp product (wild-type *p53* allele) (Marino et al., 2000).

### Image capturing and analysis

Histological slides were digitised on a LEICA SCN400 scanner (LEICA UK) at 40× magnification and 65% image compression setting, and images were stored on Slidepath Digital Image Hub (Leica Microsystems). Fluorescent images were acquired on a ZEISS LSM710 META confocal laser scanning microscope and processed with the ZEN software.

X-Gal-stained frozen sections were captured prior to counterstaining on an Olympus MegaView III digital camera, attached to a Zeiss Axioskop and then counterstained with Nuclear Fast Red, and images were digitised on a LECIA SCN400 scanner.
